# Interdisciplinary lower extremity reconstruction in peripheral artery disease with AV-loops and latissimus dorsi flaps

**DOI:** 10.1515/iss-2025-0032

**Published:** 2025-09-23

**Authors:** Simon Reß, Anja M. Boos, Alexander Gombert, Christian Uhl, Benedikt Schäfer, Lara Lingens, Astrid Bülow, Heide Delbrück, Frank Hildebrand, Justus P. Beier

**Affiliations:** Department of Plastic Surgery, Hand Surgery – Burn Center, University Hospital RWTH Aachen, Aachen, Germany; Department of Vascular Surgery, University Hospital RWTH Aachen, Aachen, Germany; Department of Orthopaedics, Trauma and Reconstructive Surgery, University Hospital RWTH Aachen, Aachen, Germany

**Keywords:** free flap, lower extremity, arteriovenous loop, microsurgery

## Abstract

**Objectives:**

Extensive soft tissue defects of the lower leg can be effectively addressed by transplantation of various free microvascular flaps. A fundamental requirement for successful free microvascular flap coverage is the availability of adequate recipient arteries and veins. In cases where vascular conditions are compromised, such as in peripheral artery disease (PAD), achieving effective defect coverage through free microvascular flap transplantation poses significant challenges. One potential treatment option is the creation of an arteriovenous (AV) - loop to facilitate the attachment of a free flap.

**Methods:**

Our study comprises patients receiving AV-loops with free latissimus dorsi flaps between 2019 and 2021. A retrospective review was performed of all patients who underwent this operative procedure with a minimum follow-up time of 3 years. We conducted pre- and intraoperative flow measurements with ultrasound. Flap survival, autonomisation and patient satisfaction was analyzed clinically, with thermography and the Freiburg Index of Patient Satisfaction (FIPS). We focus on identifying which patients benefit from this approach, as well as exploring limitations related to the severity of peripheral arterial occlusive disease (PAOD).

**Results:**

Our study showed that this procedure can be used to cover defects safely and with a low flap loss rate, even in highly compromised vascular situations. It was also shown that even in PAOD, permanent patency of the pedicle vessel is not essential for the flap. In this study, with its associated limitations, we were unable to establish a reliable correlation between preoperative and intraoperative flow measurements and flap loss in the two-stage procedure we chose. Despite the high physical and psychological stress on the individual patients, the majority were satisfied with the procedure and the result.

**Conclusions:**

Defect coverage of the lower extremity using a loop and free muscle flap is a safe, reliable procedure with a high patient satisfaction.

## Introduction

Defect coverage of the lower extremity in the presence of peripheral arterial disease (PAD) and the typically associated comorbidities such as diabetes mellitus, hypercholesterolemia and nicotine abuse [1] represents a reconstructive challenge and is associated with a significantly higher failure rate than in healthy vessels. In a retrospective study, A. Pekcan was able to show that patients with PAD have a significantly higher risk of partial or complete flap loss on the lower extremities compared to a group with a low risk of PAD [2]. In the severe form of PAD the peripheral arterial occlusive disease (PAOD) with a critical circulatory situation of the lower extremity, the 1–year amputation rates are between 15 and 20 % and the 1-year mortality rates between 15 and 40 % according to a study conducted in 2020 [3]. Particularly after traumatic tissue loss or the need for open fracture treatment, a significantly higher risk of amputation can be assumed if PAD is also present. Various concepts for preventing amputation have been developed and described for defect coverage on the lower extremity in the event of a poor connection situation. As early as 1982, Threlfall described successful defect coverage on the lower extremity with microvascular connection to an arteriovenous (AV)-loop in a case report [4]. Subsequent studies have shown that the AV-loop technique is a safe and successful procedure [5], [6], [7]. Smaller studies showed no difference in the use of venous bypass grafts or the establishment of AV-loops as a connection option [8]. A larger study dealing with the connection situation with regard to microvascular head and neck reconstruction was able to show a significant advantage in favor of the AV-loop approach [9]. Another method involves utilizing arterial branches that are typically unaffected by atherosclerotic disease. The medial sural artery and its distal branches have been effectively used as salvage recipient vessels in lower limb reconstruction due to their relative resistance to peripheral arterial disease (PAD) changes [10]. Studies by Baliarsing et al. have demonstrated the reliability of the medial sural artery in complex post-traumatic reconstructions [11], while Smith et al. emphasized the use of distal medial sural vessels as dependable recipient sites in free tissue transfer [12]. Additionally, Morelli Coppola et al. reported successful application of the medial sural neurovascular pedicle for functional latissimus dorsi free flap transfer in the posterior leg compartment [13]. This approach offers an alternative when traditional recipient vessels are unavailable. For defect coverage in the lower limb and specially in the presence of osteomyelitis, the use of a muscle flap is probably also described as the first choice for historical reasons [14], 15]. Recent multi-center outcome analyses showed no significant difference in the choice of muscle or tissue flap [16]. The question of whether the loop with flap connection should be performed in one or two stages cannot be conclusively clarified at the present time, even if there are indications of an advantageous course with a one-stage procedure [17]. Our retrospective case series focused on patients receiving defect coverage at the lower extremity with free latissimus flaps and AV-loops. An interdisciplinary concept was developed together with the Department of Vascular Surgery for flap coverage on the lower extremity in PAD patients to repair a defect otherwise requiring amputation. We chose a two-stage AV-loop approach and performed the final defect coverage using a latissimus dorsi muscle flap. Additionally, we conducted pre- and intraoperative flow measurements with ultrasound on the vessels to gain a better understanding of the changes in blood flow in the loop model at the individual measurement points and thus possibly obtain a predictive forecast regarding the outcomes. In selected patients thermography of the flap and the surrounding skin areas was conducted. Finally, we looked at the long-term course after reconstruction and patient satisfaction. Our results show that despite the high personal burden for the patients, this is a safe and long-term treatment which is overall associated with high patient satisfaction.

## Methods

In our retrospective study, we included all patients of our department from 2019 to 2021 who underwent lower extremity reconstruction and who received a latissimus dorsi flap connected to an an av-loop. Patients treated after 2021 at our department where not included to facilitate a minimum follow-up time of 3 years after the procedure. Follow-up observations and collection and documentation of all postoperative findings presented here were conducted as part of routine clinical practice and as such were part of the medical records, reviewed for this retrospective case series and was covered by the patients signing of the “Broad Consent” and a positive ethical vote by the medical faculties Ethical Committee (EK 25–261). All patients were assessed together with the Department of Vascular Surgery, and in case of trauma related defects also with the Department of Orthopedics, Trauma and Reconstructive Surgery, prior to reconstruction. Where possible, an attempt was made by the radiologists before AV-loop surgery in order to improve the vascular situation by intervention (percutaneous transluminal angioplasty (PTA) with or without stenting).

### Interdisciplinary preparation of patients

All patients underwent CT angiography with visualization of the arterial and venous phase for precise assessment of the recipient vessels’ status. If a possible indication for interventional improvement of the vascular situation resulted, digital subtraction angiography with an attempt to recanalize was carried out by the Department of Diagnostic and Interventional Radiology first. Subsequently indication for defect coverage by means of loop creation and free flap coverage with connection to the loop was made on an interdisciplinary basis and planned as a 2-step procedure. The ipsilateral great saphenous vein as the preferred bypass material, was assessed using ultrasound by the department of Vascular vurgery and was harvested if it could be clearly visualized by duplex sonography over a sufficient length with a diameter of at least 3 mm maintained throughout its course. It had to have no signs of pathological dilation due to venous valves insufficiency. Further exclusion criteria included higher-grade stenoses, advanced post-phlebitic or phlebosclerotic changes, and occluded or thrombotic vessels. In each of the 12 patients presenting for defect reconstruction and being evaluated for an AV-loop in our department within the given period of time, a great saphenous vein graft length of sufficient length and required quality could be obtained.

### Surgical protocol

Depending on the cause of the soft tissue defect, several operations were carried out in advance together with colleagues from orthopedics and trauma surgery until aseptic wound conditions were achieved (with ongoing antibiotic therapy in the case of a preoperative septic wound situation or osteomyelitis). The soft tissue defect was then preconditioned using topical negative pressure (TNP) therapy. After sufficient wound conditioning, the loop was established. In all patients, the loop was created at the level of the P3 segment of the popliteal artery. For the loop vessel a great saphenous vein graft from the contralateral side was harvested. This was anastomosed in a U-shape between the artery and the popliteal vein end to side, considering the direction of flow. After creating the loop it was placed underneath the surrounding intact soft tissue and skin envelop as close to the planned anastomosis area as possible, ensuring a sufficient vital tissue cover. The wound itself was temporarily covered with TNP therapy until final flap transplantation. Daily clinical and duplex sonography checks of AV-loop perfusion were carried out.

In three patients thrombosis in the AV-loop was detected prior to free flap transplantation at day 2, 4 and 11 respectively, after AV-loop operation. Successful intervention was performed in each case. The transplantation of the free muscle flaps did not take place earlier than 6 and 14 days later than the AV-loop was established. In the case of the loop revision, the connection of the flap was postponed by a few days. In one patient, definitive arthrodesis of the ankle was performed after the AV-loop had been established and two days before flap coverage in order to prevent possible colonization of the inserted implant material.

**Figure 1:**
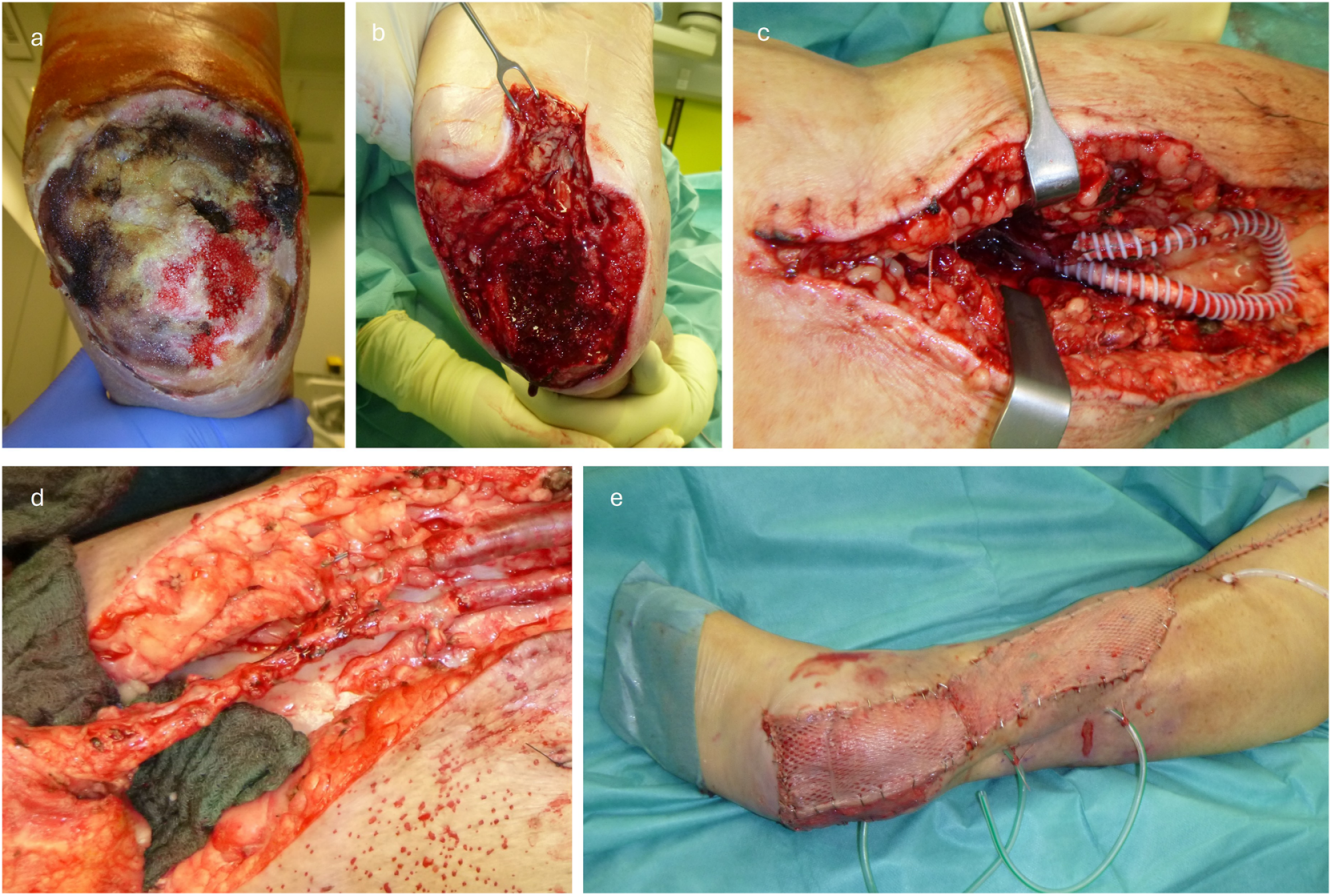
Exemplary demonstration of the surgical procedure for a) initial defect, b) after debridement c) after loop placement (enclosed by PTFE prosthesis for compression protection), d) after flap anastomosis and e) after final split skin grafting on the flap.

After harvesting the latissimus dorsi flap, the AV-loop was divided at the apex and a venous and arterial leg was established. The thoracodorsal pedicle vessels were connected in an end-to-end fashion, taking into account the direction of flow to the respective arterial and venous leg. In addition to the Doppler sonographic flow measurement through the arterial and venous leg of the AV-loop, the flap perfusion was assessed using ICG as described previously [18]. After ensuring sufficient inflow and outflow, the flap was fitted and fixed. The final covering of the muscle flap was performed using split skin graft harvested from the thigh (Figure 1). Postoperative follow-up of the patients was performed according to a standardized flap protocol with regular clinical flap checks and flow monitoring with the handheld Doppler device.

### Intraoperative flow measurement by transit-time flowmetry

Intraoperatively patients in vascular surgery routinely receive assessment by intraoperative transit-time flowmetry to determine blood flow volume. This ultrasound-based measurement method determines the transit time via a probe placed in direct vicinity of the vessel wall using the Doppler effect. This is proportional to the blood flow volume. Previous studies have confirmed this relationship with high correlation [12], 13]. Flow measurement was performed with the Medistim VeriQ™ flowmeter and the transit time flow measurement (TTFM) probe (Medistim, Oslo, Norway). The flow measurements were carried out at pre-defined timepoints: the first measurement was taken immediately after the loop was established at both branches, where an arterial and a venous branch were defined according to the direction of flow as part of the routine vascular surgery procedure. The second measurement was taken before the loop was cut (day 6–14 after establishment) also at both legs. Additionally, measurements were taken at the arterial and venous legs after exposing the pedicle. This was conducted again after anastomosing the flap to the loop and maintaining a flap inflow time of 10 min. The measurements were carried out under constant blood pressure conditions of 120/80 mmHg. For the measurement, the probe was placed and after 15 s, upon a steady pulse wave and stable signal, the measurement was taken.

The statistical analysis of the flow measurements was performed using GraphPad Prism. For analysis, we chose the paired t-test as a statistical method to compare two related measurements taken from the same subject. The measurement values for individuals before and after intervention, as well as early and later stages of loop establishment, were recorded and compared.

### Infrared thermal imaging

As part of our routine long-term clinical follow-ups for flap patients, we use non-invasive thermography for additional assessment of the postoperative course and flap perfusion. We used the dynamic thermography for non-invasive assessment of the reheating patterns and the indirect assessment of blood flow according to our previously described protocol. A publication from 1995 already provided supportive assessment of blood flow with thermography in the context of reheating patterns in a venous flow-through and pedicled venous flaps mouse model [19]. In a large review by Ramirez-Garcia Luna from 2022, several benefits of thermography in visualizing perfusion patterns were demonstrated [20]. Thermal images were obtained using the VarioCam^®^ HDx head 600 (InfraTec GmbH Infrarotsensorik und Messtechnik, Dresden, Germany). The VarioCam^®^ contains a uncooled microbolometer focal plane prray detector, with a 640 × 480 format (IR pixels) a spectral range from 7.5 to 14 µm. The temperature resolution at 30 °C is up to 0.03 K. The camera has a scene range temperature of −40 to 600 °C. The camera was placed on a Tripod and connected to a notebook via network cable for visualization of real time thermography. The investigation was conducted in a climate-controlled room of our outpatient clinic as part of our routine follow-up assessment in reconstructive patients at a constant room temperature of 21 °C. The patients had already been in the room for at least 30 min for acclimatization at the time of the investigation. First, images were taken of the legs in a side-by-side comparison. The dynamic recordings were made while lying down. For this purpose, the area of interest was initially cooled with a common 10 °C pre-cooled gel pad (40 × 60 cm) for 90 s. Subsequently, continuous image documentation was carried out over 10 min with one image every 2 s. The image analysis was made with IRBIS^®^3 professional Software. The graphically displayed temperature range was defined individually for each patient for better visual representation and set constant over the entire time with the patients surrounding skin temperature serving as a reference.

### Freiburg Index of Patient Satisfaction (FIPS)

Patients who were available for follow-up and could be evaluated regarding patient satisfaction with the surgical procedure performed using the Freiburg Index of Patient Satisfaction (FIPS) [21]. The FIPS is a questionnaire to assess treatment-related patient satisfaction after surgery and interventional procedures. It includes four questions regarding personal experiences of the operative procedure (how stressful it was, the time to recover, estimation of treatment success, “would they do it again”) and an additional overall rating by the patient. In this context, a good FIPS score of 2.8 (SD=0.59) was observed on a scale from 1=excellent to 6=very poor.

## Results

Based on the evaluation of the available medical records, review of external findings, and as far as possible, follow-up of the patients, we conducted a retrospective evaluation of 12 patients from 2019 to 2021. All 12 patients were treated in an interdisciplinary approach with a combination of AV-loop serving as arterial and venous recipient vessels followed by transplantation of a free (split) latissimus muscle flap. All patients had a soft tissue defect of the lower extremity requiring a large surface covering flap. Underlying cause was soft tissue infection (3 patients), post-traumatic osteomyelitis (6 patients), acute trauma (2 patients) and skin tumor resection (1 patient). In all patients, the recipient vessel quality and/or availability at the lower extremity was significantly compromised due to advanced arteriosclerosis (n=10) or traumatic vessel lost (n=2). The age of the patients (10 men, two women) ranged from 44 to 83 years with a median age of 66 years. Prior to the planned surgical treatment, all patients underwent radiological and vascular surgical assessment with the question of improving the vascular situation. In 10 patients, a higher-grade PAOD was already known from their medical history or based on previous findings and could be confirmed in the further course, leading to the indication for AV-loop creation due to the PAOD-related compromised vascular situation. In one patient, the loop was placed secondarily after primary flap loss and with previously undetected symptoms of venous congestion. In one female patient (patient no. VII), AV-loop was necessary due to a traumatic multi-level injury of the two main vascular axis of the lower leg with no signs of PAD. A CT angiography (n=12) was performed in all patients. If there was potential for improvement, a digital subtraction angiography (DSA) (n=6) was performed with possible intervention (n=4) three patients received a recanalization with percutaneous transluminal angioplasty (PTA). The intervention was unsuccessful in one patient (VI). In three patients, a surgical intervention was performed prior to AV-loop creation based on pathological CTA results: two patients received a bypass (1 femoropopliteal, one popliteo-fibular), one patient received a femoral bifurcation thromboendarterectomy procedure. The following table gives an overview of all patients’ PAOD staging and initial treatment prior to AV-loop and flap procedure (Table 1).

**Table 1:** Overview of all patients’ PAOD staging and initial treatment prior to AV-loop and flap procedure.

Patient no.	Gender	PAOD stage (acc. to fontaine)	Pathological CTA finding?	DSA	Inter- vention?	Type of intervention
I	♂	IIb	+	–	+	Femoral bifurcation TEA
II	♂	III	+	+	+	Recanalization of the anterior tibial artery
III	♀	III	+	+	+	Recanalization of the anterior tibial artery
IV	♂	III	+	+	–	
V	♂	–	+	–	+	Popliteo-fibular bypass
VI	♂	III	+	+	+	Unsuccessful intervention
VII	♀	–	–	–	–	
VIII	♂	III	+	–	–	
IX	♂	III	+	–	–	
X	♂	III	+	+	+	Recanalization of the superficial femoral artery
XI	♂	III	+	–	+	Femoropopliteal bypass
XII	♂	IIb	+	+	–	

With chosen a two-step procedure, in two patients, a revision of the loop had to be performed prior to scheduled flap transplantation due to occlusion. In those patients flap loss would have been a significant risk while the 2-step approach ensured AV-loop patency before performing flap transplantation. In one female patient, the loop revision was performed during flap placement as there was a significant decrease in flow volume sonographically before separation, despite a clinically inconspicuous AV-loop.

Transplantation of free (split) latissimus dorsi flaps was performed in average after 9 days (12–6 days; mean 8.8 SD 1.86) following AV-loop procedure. In three patients, early flap revisions were necessary, due to thrombosis of the AV-loop (2x arterial a 1x time venous thrombosis) which ultimately proved to be frustrating. In the case of the venous thrombosis the AV-loop could be salvaged and a successful secondary flap transplantation (rectus abdominis flap) was performed 7 days after AV-loop revision. In one more patient, flap loss occurred after4 weeks. The wounds were converted into a chronic wound (n=2) with the establishment of a permanent fistula in the case of chronic osteomyelitis (n=1). In one patient, amputation of the corresponding lower extremity was performed during the initial stay.

**Figure 2:**
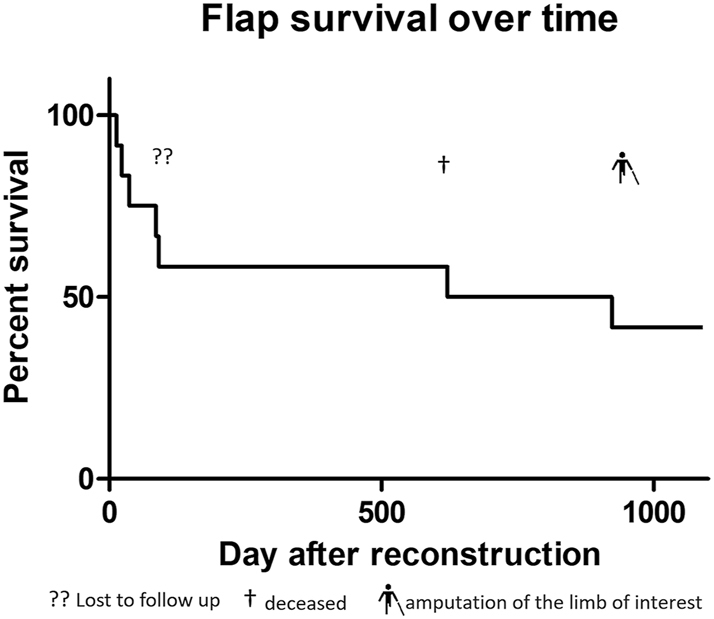
Flap survival curve in percentage over 3 years.

In a follow-up period of 3 years, four patients could be directly monitored, with one patient undergoing above-knee amputation after 2.6 years due to osteomyelitis of the heel with still well-perfused flap. Two patients were contacted by phone, confirming that the flap remained *in situ*. For two patients, no data after the date of discharge could be obtained (lost to follow-up) (Figure 2).

**Figure 3:**
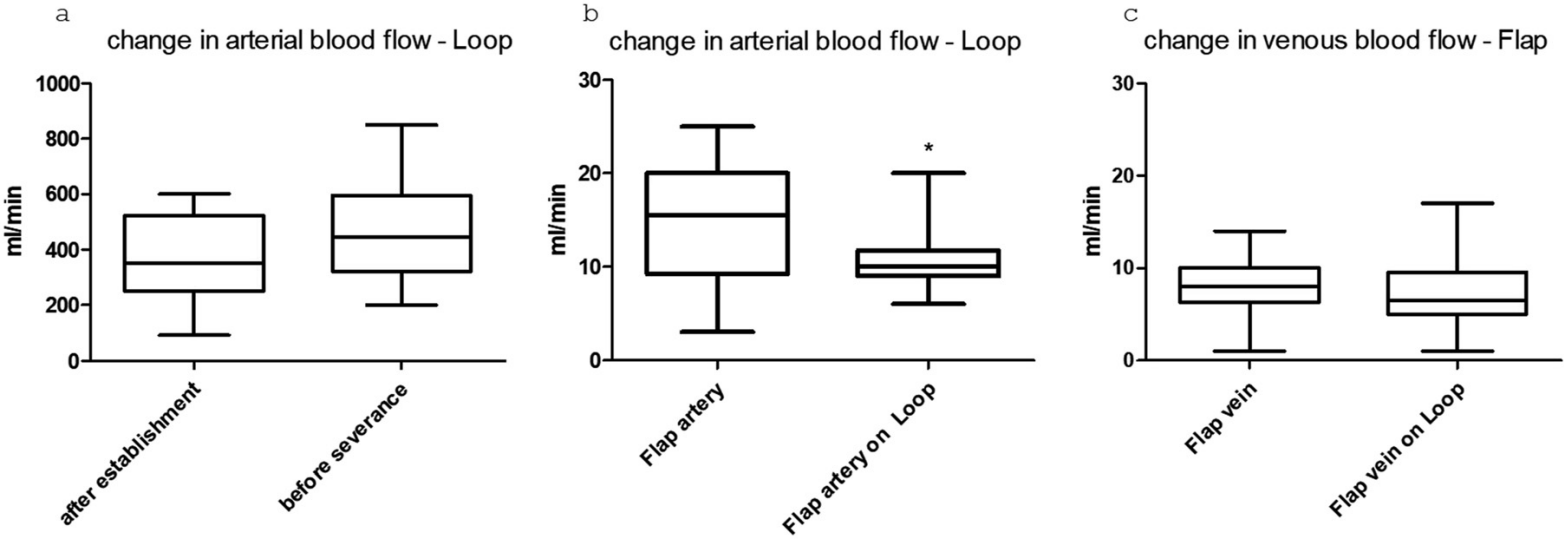
Boxplots showing the flow changes in the vessels after a) loop placement and before transection. b) In the arterial flap vessel before placement and after connection to the loop (*=p value<0.05), and c) in the venous flap vessel before and after connection.

In the evaluation of the preoperative and intraoperative measured blood flow values, no dependency could be observed between initial blood flow, changes in blood flow, flap size, and flap survival in our group. As expected, a tendency to increase in the flow rate of the loop was observed after placement and before flap closure in the sense of the loop “establishment” (p=0.16, n. s.). After connecting the muscle flap to the loop, a significant (p=0.02) drop in arterial flow in the flap vessel was observed after connecting the flap to the loop compared to the physiological flow rate before disconnection (t=10 min after connection) (paired t-test). In the venous limb, no significant difference was observed between the two measurement points (p=0.9 ns) (Figure 3).

**Figure 4:**
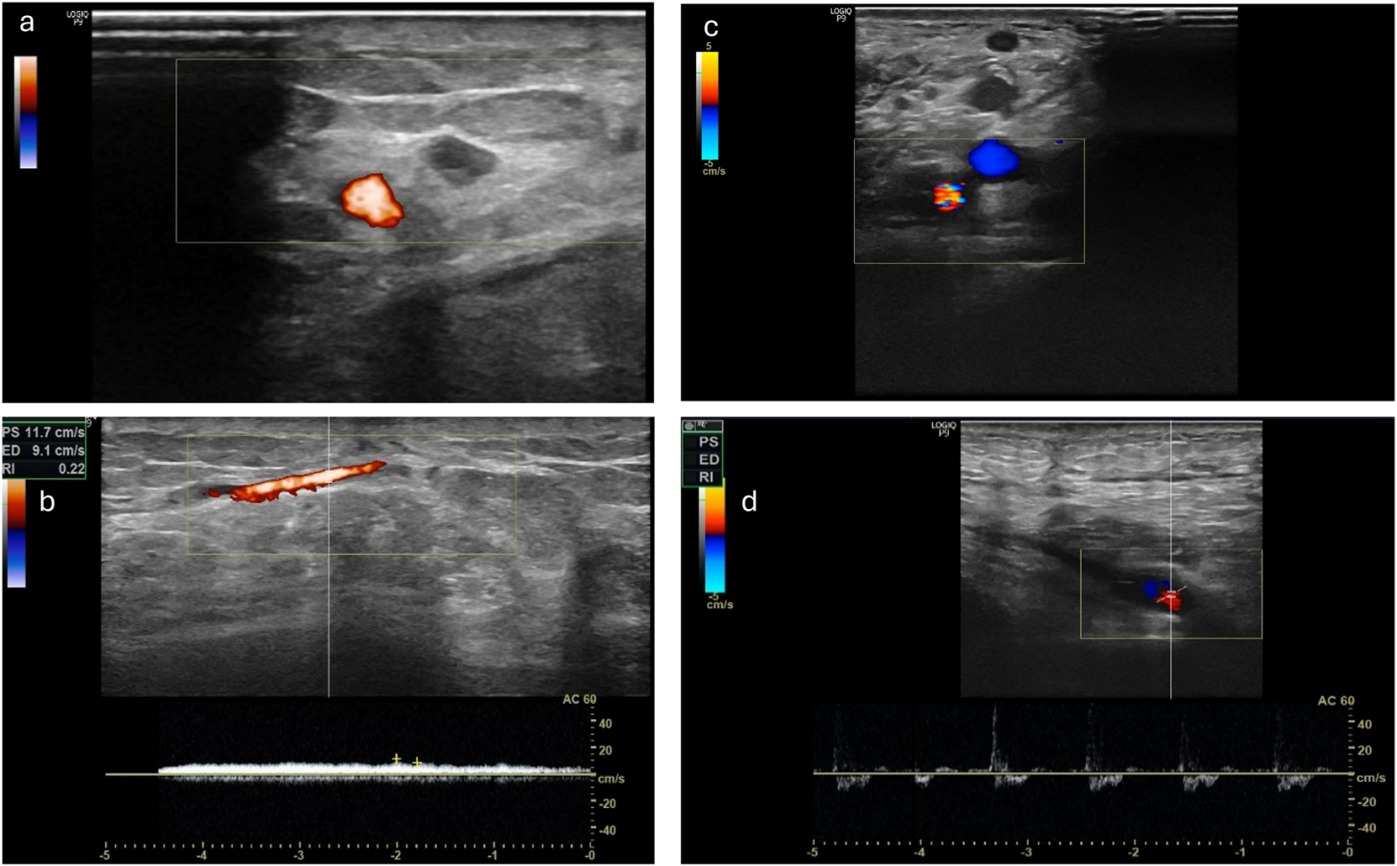
Left side a) and b) the venous leg of the loop is still patent, with a venous flow signal in the CW Doppler ultrasound while arterial leg is occluded (patient VII) right side c) and d) the AV-loop is still patent and both legs are still perfused with an arterial flow signal in the CW Doppler (patient XII).

Further monitoring of the patients took place as part of the planned regular follow-ups. Here, the clinical assessment of the flap and the photographic documentation of the transplanted flap were carried out. This enabled the retrospective evaluation of the patients regarding successful transplantation and limb preservation. In four patients, an assessment of the pedicle using Doppler sonography could additionally be performed. Overall, the procedure shows a critical interval in the first month. In the further course, a stable flap situation is observed in the patients we evaluated. One patient died due to her underlying conditions after 621 days with the flap still in place. In one patient, there was an indirect flap loss after 923 days following amputation of the corresponding limb due to osteomyelitis of the heel, without any causal connection to the procedure we performed. Sonographically, an occluded pedicle was observed in two patients with a vital flap. In one patient, only the venous outflow could be demonstrated through the former loop. In one patient, open pedicle vessels were sonographically shown after 1,168 days (Figure 4). Regarding flap assessment, no significant differences were observed between the patients with patent, partially occluded or completely occluded loop.

**Figure 5:**
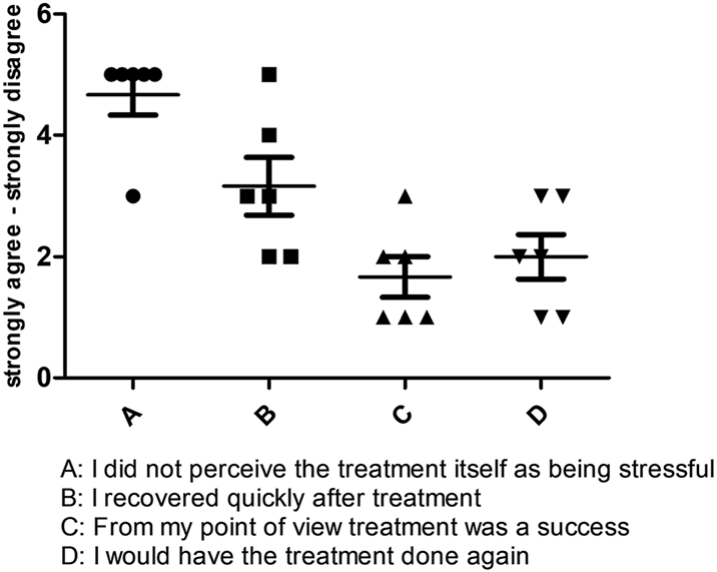
Graphical representation of the responses of all six patients in the FIPS.

Six patients were available for follow-up. The result shows a good overall FIPS score of 2.8 (SD=0.59). It is noteworthy that the surgical treatment was perceived as rather stressful by all patients with a bad score of 4,7 (SD=0,81). The answer regarding how quickly they recover from the operation procedure are with the greatest distribution and appear in the median neutral with a Score of 3.6 (SD=1,16), most of the patients in our cohort agree with the question if they would undergo this procedure again with very Good Score of 1.6 (SD=0.82) and describe the treatment as successful with a good Score of 2 (SD=0.89) (Figure 5).

**Figure 6:**
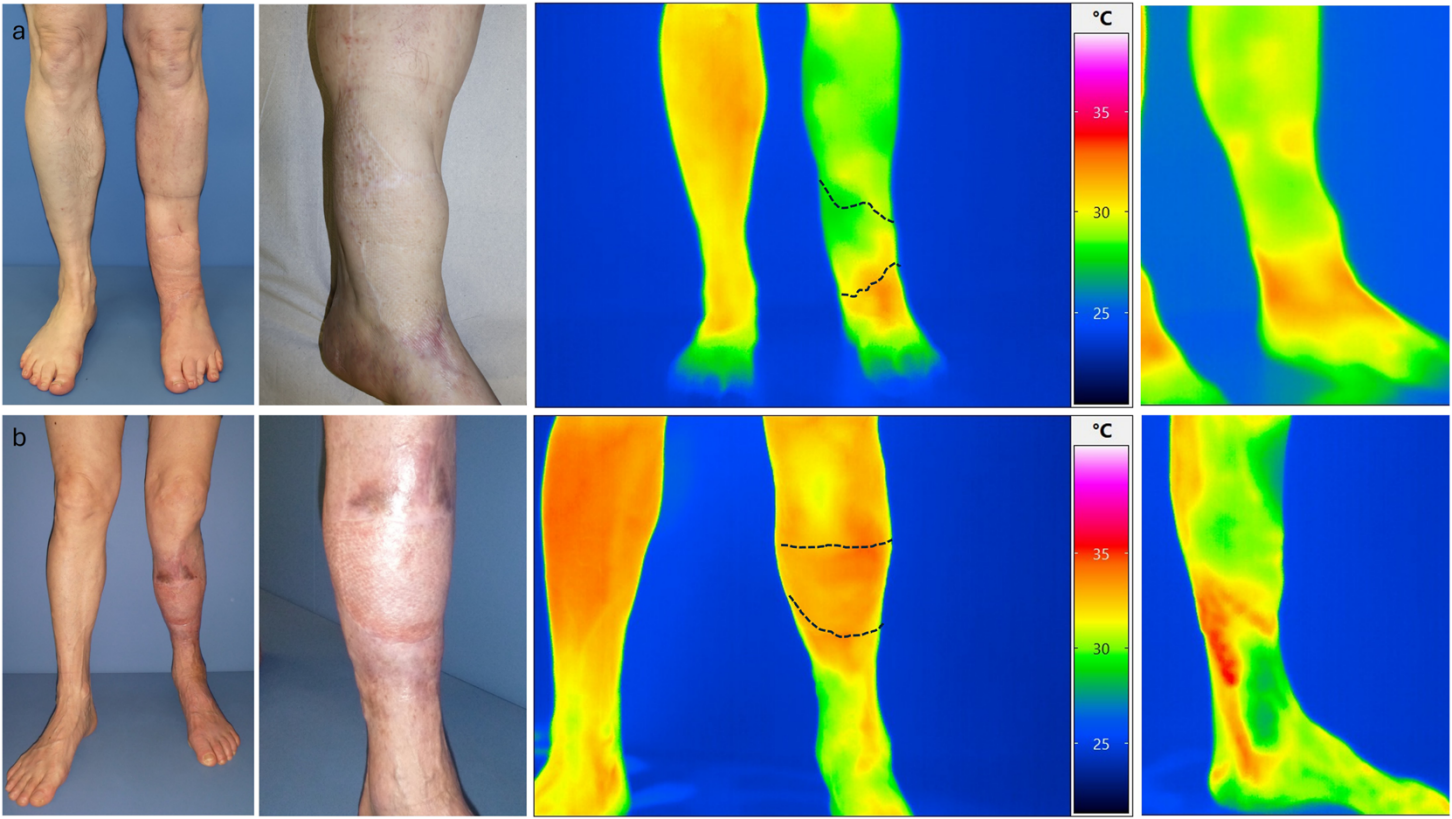
a) Upper row images of patient XI (occluded loop) and b) lower row patient XII (open loop) with native image and thermographic images in standing position.

**Figure 7:**
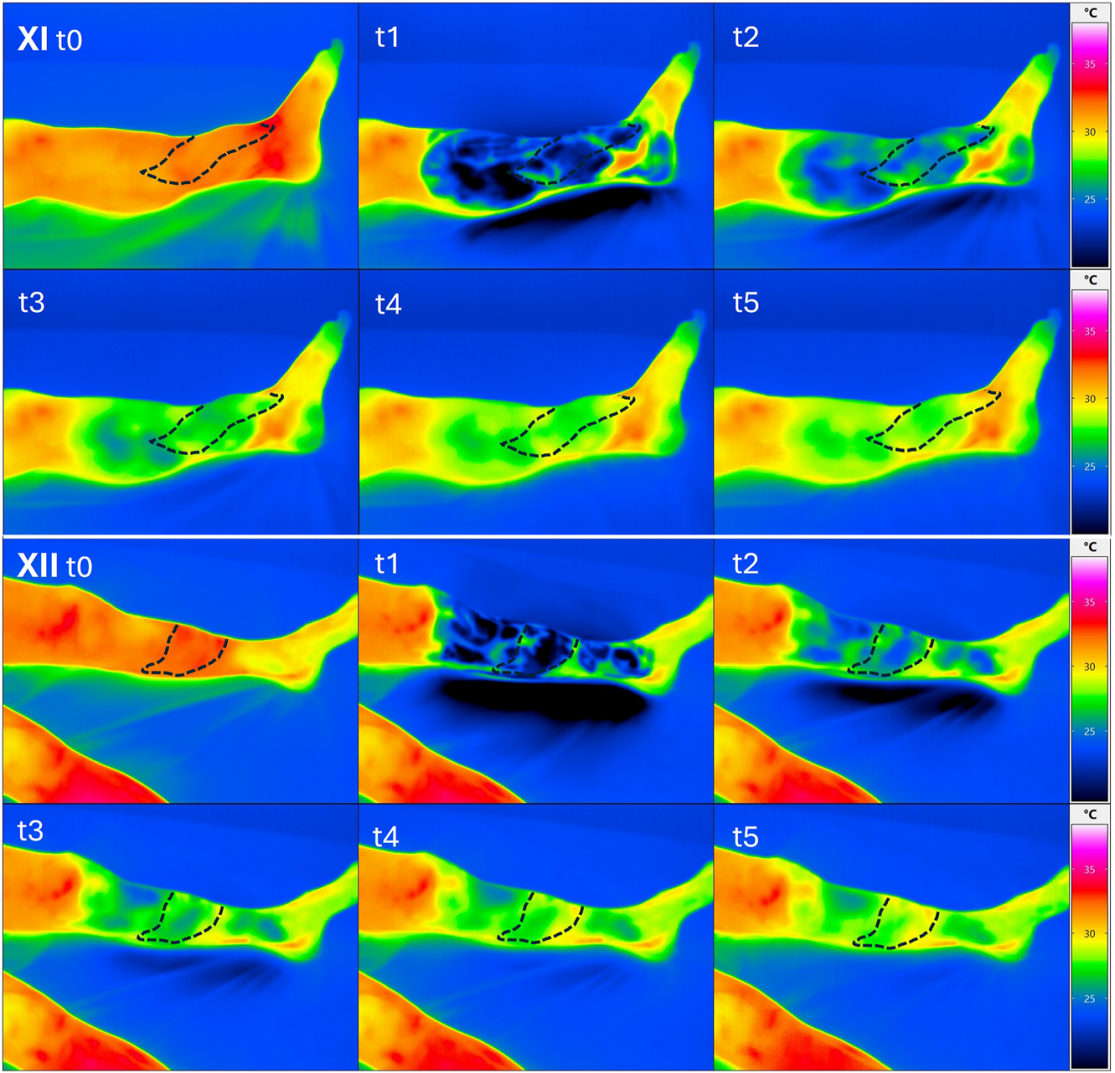
Upper image box: dynamic thermographic images of patient XI (closed loop) at the beginning (t0) after cooling for 90 s (t1) and subsequently after 2.5 min (t2), after 5 min (t3) after 7.5 min (t4) and after 10 min (t5). No difference in warming of the flap area compared to the proximal soft tissue with rewarming of the flap beginning from distal. Lower image box: dynamic thermographic images of patient XII (open loop) at the beginning (t0) after cooling for 90 s (t1) and subsequently after 2.5 min (t2), after 5 min (t3), after 7.5 min (t4) and after 10 min (t5). Visibly faster warming in the area of the inserted flap (middle tibia) compared to the distal and proximal tissues.

We performed dynamic thermography exemplarily in two patients at a late observation time point after 3 years. Here, we selected one patient with sonographically completely occluded loop and one with open loop. Already in the static images, we were able to show a significantly improved local perfusion in the flap area in the case of the open loop despite clinically unremarkable local findings. Additionally, the PAD-related compromised perfusion situation was shown in comparison to the opposite side. Interestingly, the flap perfusion in the occluded loop occurred despite PAD due to a distally emphasized autonomization of the flap (Figure 6). Using dynamic thermography, a faster warming of the flap area compared to the surrounding tissue was also observed in the case of the open loop (Figure 7). In the case of the occluded loop, as already shown in the static image, a distally emphasized warming of the flap was observed (Figure 7).

## Discussion

**Figure 8:**
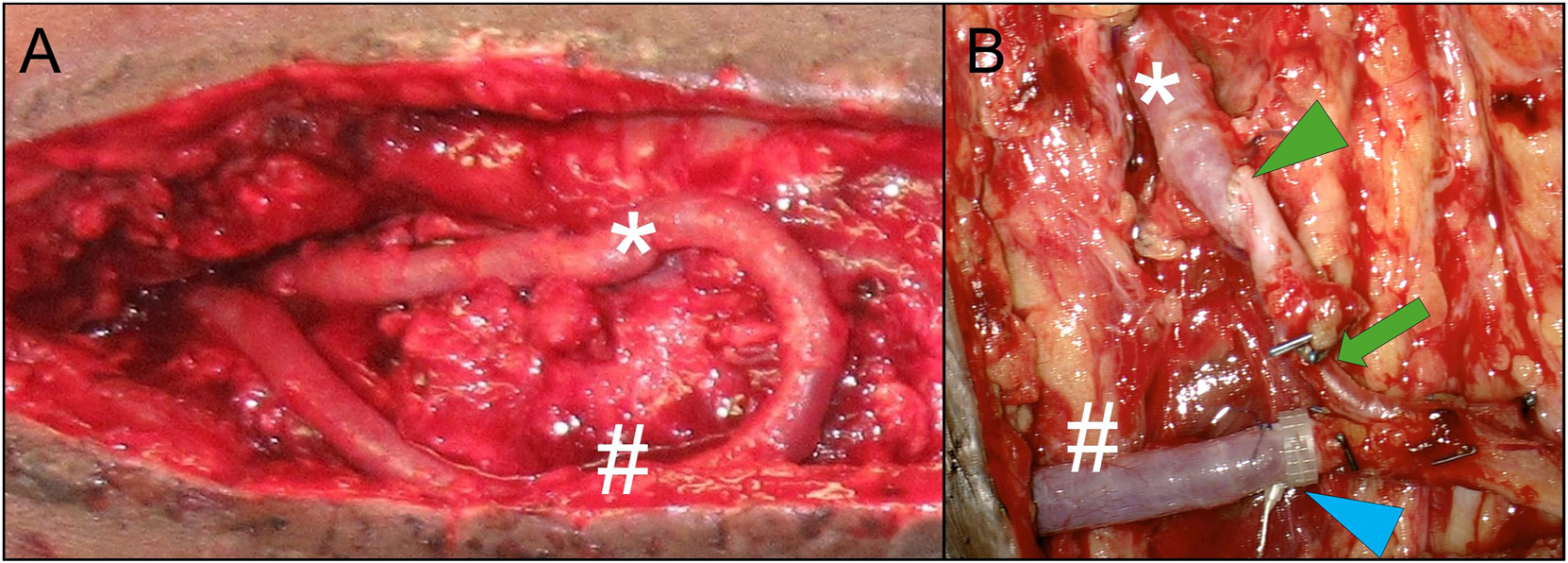
A) AV-loop exposure during flap procedure *in situ*, prior to dissection into arterial (*) and venous (#) leg B) after dissecting the AV-loop into arterial (*) and venous (#) leg and anastomosis of a free latissimus dorsi muscle flap: for the arterial anastomosis (green arrowhead), compensation of mismatch between arterial loop leg and subscapular (pedicle) artery is achieved by asymmetrical beveling of the two vessel endings (less beveling of the larger diameter AV-loop artery than the smaller diameter flap pedicle artery). Further tapering of the arterial inflow vessels occurs at the transition into the smaller caliber thoracodorsal artery (green arrow); venous anastomoses (blue arrowhead) is performed via 4.0 mm flow coupler device (Synovis Micro Companies Alliance, Inc., AL, USA).

The amputation of extremities not only leads to a social stigma but also, particularly in the case of the lower extremity, to massive functional limitations with a loss of independence. This is exacerbated in the presence of comorbidities and significantly impaired compensation mechanisms. Furthermore, in the presence of PAOD, the risk of amputation is significantly increased [22]. Even a unilateral amputation of the lower limb leads to a significant restriction, which increases considerably in the presence of comorbidities. Studies that included all subjects undergoing a lower limb amputation reported that less than half of the elderly population achieved a household level of prosthetic mobility [23]. It can be assumed that the level of immobility in our group is even higher due to the concomitant diseases. Even with a significantly reduced function, the benefit of a lower extremity that can only be used to a limited extent for movement in the home environment or for repositioning the patient should not be underestimated. Since a classic defect coverage is often not possible for this patient group due to a lack of connection possibilities, the loop technique offers a good and often the only option for preserving the extremity. With significantly prolonged hospital stays, increased surgical and anesthesia risks, and a significantly longer duration of the procedure compared to amputation, this method must be weighed against the existing limitations and the patient’s wishes. Also a higher risk of flap loss is reported in the present of arterial reconstruction (AV-loops or bypass grafts) with 79 % successful flaps initially in the study from U. Rother with 100 patients [24] and a follow-up with 59 % after 1 year. Our study shows similar results with an initial success rate of approx. 75 % and a positive follow-up of 58 % after one years, 50 % after two years and 42 % after 3 years caused by death and amputation without a causal relationship to the flap but based on the underlying diseases. One of the main reasons for loop revision and flap loss in the early phase was found triggered by thrombosis of the loop. Several reasons of this problem and its causes have already been described in the literature. One of the possible reasons of thrombosis may be an endothelial reaction described by Y. Tanaka in an animal model caused by shear stress at the arterial anastomosis region and the outer luminal surface of the curved section [25]. Also the radius of the curved section from the loop might have an influence on the development of thrombosis. The role of shear stress is also described in the study form L.D. Browne with a higher amount in “AV fistula” (=“AV-loop” in this context) with smaller radius [26]. It could also be shown in the animal model that a significant neointimal hyperplasia and venous stenosis developed by 28 days at the graft-vein anastomosis [27]. Another mechanism described in the literature is based on the difference in size between the anastomosed vessels. J.J. Monsivais was able to show in the animal model that a significantly increased occlusion rate occurs from a ratio of over 0.75:1 between graft and artery [28]. Also, J.R. Harris described a prone to thrombus formation and subsequent free flap failure with size mismatch vein grafts [29]. Based on these previous studies and due to multiple AV-loop revisions before final reconstruction it seems useful to wait for an establishment of the loop to be able to aim early thromboses due to changes in flow, but without extending the time between the placement of the loop and the connection of the free flap too long to prevent possible shear stress damage. It can also be useful in a two-stage approach to completely remove the curved section of the loop at the point of maximum possible endothelial damage. Also many techniques are described for microvascular anastomosis of vessels with different size discrepancy [30]. Exemplary shown in Figure 8, a possible solution for dealing with size mismatch between the AV-loop vessels and the flap’s pedicle vessels. Change in arterial blood flow over time in the loop is also shown in our studies before flap anastomosis are measurable but not significant. Significant alteration between arterial blood flow in the flap before and after connection to the loop are observed 10 min after connection (p=0.023). An adjustment to the initial value can be assumed over time but cannot be validated with our model at a later timepoint. These measurement results support the hypothesis that the early flap loss is also caused by a relatively high vascular resistance of free flap after transplantation. This risk factor could be demonstrated in a small study [31]. Our data could not derive any predictive value with regard to the pressure increase (= flow rate increase). The alteration of venous blood flow before and after flap anastomosis to the loop is nearly unchanged. Differences in the measurement of arterial inflow and venous outflow are most likely to be multifactorial with natural lymph loss and minor blood loss via the flap surface, as well as measurement inaccuracy in clinically non-congested flaps. As described above, no risk parameters regarding flap survival or long-term success could be derived from the flow measurements. With four flaps that were sonographically checked for loop patency after 3 years, we were able to show that complete autonomisation of the flap can also occur in PAOD patients. This is consistent with the results described in the literature [24]. Since we had early flap losses up to day 37, we assume that the times described in case reports [32] and in the animal model [33] of about 2 weeks for flap autonomisation in the presence of a PAOD are not comparable. The steal phenomenon in the lower extremity reconstruction, mainly following bypass surgery, is mentioned in some case reports [34], 35]. This could theoretically also be triggered by the AV-loop when being performed as a two-stage procedure. However, in none of the patients we operated this problem was clinically observed.

In the static thermographic flap assessment of two patients, one with a patent AV-loop and one with an occluded AV-loop, a locally higher heat signature was observed in the previous defect area in the patient with the patent AV-loop. We do not assume a measurement error due to the different surface properties (split skin vs. physiological skin), as there was no detectable difference to the surrounding tissue in comparison to the patient with the occluded AV-loop. This was also shown in the dynamic thermography in which there was a faster detectable central temperature increase in the flap in the patent loop situation. In comparison to the occluded situation, a temperature increase coming from distal to the flap boundary in the sense of a distally emphasized flap autonomisation was seen in the occluded loop. Based on the thermographic results and the assumed additional effects of the loop model, it can be hypothesized that this approach also leads to an improvement in the overall perfusion of the corresponding extremity and to a reduction in ischemia in individual patients. Three flap losses in the cohort we observed led to amputation or chronic wound situations without satisfactory secondary healing. In all successful defect coverages, satisfactory mobility could be preserved, with only one known complication at the donor site due to scar tension, which could be resolved secondarily. The primary limitation of this study is its retrospective, single-center design coupled with a small sample size of 12 patients, which restricts the generalizability of the results and diminishes statistical power. Although intraoperative flow measurements offer valuable functional insights, their application varied among patients and over different timepoints, and they are influenced by operator dependency. Additionally, long-term follow-up was not possible for all patients, with two individuals lost to follow-up. The absence of a control group limits direct comparison with other reconstructive strategies, such as single-stage reconstruction or bypass-based approaches. Furthermore, the correlation between perfusion measurements and clinical outcomes could not be conclusively validated, and no predictive threshold for flap failure was established. These limitations highlight the necessity for prospective, multicenter studies employing standardized evaluation protocols to confirm and expand upon these findings. In spite of the limitation, we have a high consistency within the performed surgical procedure, for a procedure that is rarely carried out. Considering these limitations our data suggest that after a prolonged critical early phase, despite a highly compromised vascular situation, the AV-loop plus latissimus flap can result in a lasting stable soft tissue situation. As also shown in another study [24], our data suggest that even in critical blood flow situations in the surrounding tissue, sufficient autonomisation of the flap occurs over time. Our evaluations have shown that the AV-loop plus muscle flap is suitable as a long-term and safe approach for preserving the extremity and is associated with high patient satisfaction.
